# Photochemical Internalization of Peptide Antigens Provides a Novel Strategy to Realize Therapeutic Cancer Vaccination

**DOI:** 10.3389/fimmu.2018.00650

**Published:** 2018-04-04

**Authors:** Markus Haug, Gaute Brede, Monika Håkerud, Anne Grete Nedberg, Odrun A. Gederaas, Trude H. Flo, Victoria T. Edwards, Pål K. Selbo, Anders Høgset, Øyvind Halaas

**Affiliations:** ^1^Department of Clinical and Molecular Medicine (IKOM), Norwegian University of Science and Technology, Trondheim, Norway; ^2^Centre of Molecular Inflammation Research (CEMIR), Norwegian University of Science and Technology, Trondheim, Norway; ^3^Department of Infection, St. Olavs University Hospital, Trondheim, Norway; ^4^Department of Radiation Biology, Institute for Cancer Research, Oslo University Hospital – The Norwegian Radium Hospital, Oslo, Norway; ^5^Department of Chemistry, Faculty of Natural Science, Norwegian University of Science and Technology, Trondheim, Norway; ^6^Centre for Molecular Medicine Norway, Nordic EMBL Partnership, University of Oslo, Oslo University Hospital, Oslo, Norway; ^7^PCI Biotech AS, Oslo, Norway

**Keywords:** therapeutic tumor vaccination, peptide vaccination, CD8+ cytotoxic T cells, MHC class I antigen presentation, cross-presentation, adjuvant effect, photochemical internalization, photosensitizer

## Abstract

Effective priming and activation of tumor-specific CD8+ cytotoxic T lymphocytes (CTLs) is crucial for realizing the potential of therapeutic cancer vaccination. This requires cytosolic antigens that feed into the MHC class I presentation pathway, which is not efficiently achieved with most current vaccination technologies. Photochemical internalization (PCI) provides an emerging technology to route endocytosed material to the cytosol of cells, based on light-induced disruption of endosomal membranes using a photosensitizing compound. Here, we investigated the potential of PCI as a novel, minimally invasive, and well-tolerated vaccination technology to induce priming of cancer-specific CTL responses to peptide antigens. We show that PCI effectively promotes delivery of peptide antigens to the cytosol of antigen-presenting cells (APCs) *in vitro*. This resulted in a 30-fold increase in MHC class I/peptide complex formation and surface presentation, and a subsequent 30- to 100-fold more efficient activation of antigen-specific CTLs compared to using the peptide alone. The effect was found to be highly dependent on the dose of the PCI treatment, where optimal doses promoted maturation of immature dendritic cells, thus also providing an adjuvant effect. The effect of PCI was confirmed *in vivo* by the successful induction of antigen-specific CTL responses to cancer antigens in C57BL/6 mice following *intradermal* peptide vaccination using PCI technology. We thus show new and strong evidence that PCI technology holds great potential as a novel strategy for improving the outcome of peptide vaccines aimed at triggering cancer-specific CD8+ CTL responses.

## Introduction

In addition to the well-known uses in prophylaxis of infectious diseases, vaccination also has a great potential in immunotherapy of cancers and infectious diseases. Many current vaccination protocols consist of recombinant molecules or subunits of pathogens delivered in combination with an immunological adjuvant, and protect mainly by induction of antibody-mediated humoral immune responses toward extracellular antigens (1). Similar to many pathogens, these vaccines are endocytosed by antigen-presenting cells (APCs) for lysosomal degradation and subsequent entry into the MHC class II antigen presentation pathway. This leads to activation of CD4+ T helper cells, which induce B cell and antibody-mediated immunity. Antibodies are effective against extracellular pathogens, but cannot directly access intracellular pathogens, such as viruses or intracellular bacteria, and are also largely ineffective in eliminating cancer cells. CD4+ T helper cells may help to fight intracellular pathogen infections by producing pro-inflammatory cytokines that activate macrophages to destroy intracellular pathogens or contribute to killing of diseased cells (2–5). However, vaccine strategies primarily inducing CD4+ T helper cells have been found ineffective against a variety of intracellular infections and malignant diseases.

It is generally recognized that CD8+ cytotoxic T cells (CTLs) are most effective in combating these diseases, as they are licensed to selectively eliminate infected cells as well as malignant or otherwise abnormal cells (6). A critical pre-requisite for successful CD8+ CTL activation is localization of antigens in the cytosol of APCs, from where they can be transported into the lumen of the endoplasmic reticulum to be loaded onto MHC class I molecules for subsequent presentation to CD8+ T cells. The presence of foreign antigens in the cytosol typically occurs when cells are productively infected during virus infection or immunization with attenuated live virus vaccines. Vaccination approaches aiming at CD8+ CTL activation, therefore, include the use of genetically engineered or attenuated live viruses that can enter the cytosol. Although often effective, such approaches have several severe drawbacks, such as various safety concerns for immunocompromised people, limited potential in antigen selection, lengthy development, and the generation of antibody responses to carrier viruses which often precludes repeated vaccinations (7). Many of these concerns could be alleviated by using subunit, protein- or peptide-based vaccines, if formulations could be developed which efficiently promote translocation of endocytosed vaccine antigens to the cytosol of APCs in order to achieve efficient priming of CD8+ CTLs (8).

Peptides have attractive features for use as vaccine antigens, particularly for therapeutic cancer vaccination, as they are generally non-toxic, cheap, and easy to synthesize in Good Manufacturing Practice quality. Peptides can readily be tailored for patient-specific therapy and are thus a promising approach to raise specific CD8+ CTL responses against neo-epitopes and tumor mutations found in patients (9). However, a generally low immunogenicity and difficulties in delivery to the cytosol in APCs pose hurdles for peptide vaccination strategies, perhaps accounting to a large extent for the failures in advanced clinical trials [reviewed in Ref. (10)]. Developing technologies that can make peptide-based vaccination more effective and that can translate this vaccination strategy into an efficient clinical modality, therefore remains critical.

Here, we explore an approach utilizing photochemical internalization (PCI) to enable cytosolic delivery of peptide antigens for enhancement of MHC class I presentation and subsequent CD8+ CTL priming and activation. PCI is a technology for the release of endocytosed material into the cytosol by the use of photoactive compounds (photosensitizers). The principle is based on co-endocytosis of an amphiphilic photosensitizer molecule that incorporates into endosomal membranes together with cargo material that is aimed for cytosolic delivery. Illumination activates the photosensitizer molecule and leads to disruption of the endosomal membranes by production of reactive oxygen-species and/or singlet oxygen, which releases the cargo molecules into the cytosol (11–14). Only structures very close to the photosensitizer will be affected as the effect is very short-lived and has a radius of action limited to nearby molecules. PCI has been shown to facilitate cytosolic delivery of a large variety of molecules that do not readily translocate into the cell cytosol, such as proteins, peptides, DNA, and some small molecule drugs (11, 12, 15–18). The photosensitizer used in this study is meso-tetraphenyl chlorin disulphonate, fimaporfin (TPCS_2a_), a compound that is under clinical development for local cancer treatment (19, 20). The potential of PCI using TPCS_2a_ to deliver antigens for subsequent CD8+ CTL activation has been shown for full-length ovalbumin (OVA) protein in a mouse model with adoptively transferred CD8+ T cells (21) and was shown to reduce growth of malignant OVA-producing cells (22, 23).

In this work, we studied if PCI technology may provide a novel strategy to improve effectiveness of peptide antigen vaccination. We provide a detailed characterization of the effects of PCI-mediated cytosolic delivery of peptide antigens and PCI effects on antigen presentation, survival, and phenotype of APCs. We show that PCI enhances CD8+ T cell activation *in vitro* and can prime CD8+ CTL responses *in vivo* with various cancer-relevant peptide antigens in mice. Our results demonstrate that PCI using TPCS_2a_ constitutes a powerful technology for enhancing MHC class I presentation and CD8+ CTL priming by peptide antigens and that the technology, in addition, induces an adjuvant-like immune cell activation. Thus, PCI peptide vaccination technology has the potential to be employed for vaccination strategies that aim at induction of CD8+ CTL responses.

## Materials and Methods

### Light Source and Photosensitizer

Cells were illuminated on the LumiSource^®^ (PCI Biotech, Oslo, Norway) light table, a light source designed to provide homogeneous blue light illumination with a peak wavelength of 435 nm (24). An illumination time of 1 min corresponds to a light dose of 0.81 J/cm^2^. The photosensitizer meso-tetraphenylchlorin disulphonate, TPCS_2a_ (fimaporfin) in the Amphinex^®^ formulation was provided by PCI Biotech (Oslo, Norway) (19). The Amphinex formulation contains 30 mg/ml TPCS_2a_ in 3% polysorbate 80, 2.8% mannitol, 50 mM Tris–HCl pH 8.5.

### Antigen-Presenting Cells

Immortalized C57BL/6 macrophages (B6) were generated with J2 recombinant retrovirus as described (25, 26). Primary bone marrow-derived macrophages (BMDMs) were generated by cultivating mouse bone-marrow cells for at least 5 days in medium supplemented with 20% L929 cell line supernatant (ECACC). Immature bone marrow-derived dendritic cells (BMDCs) were generated by cultivating murine bone marrow cells for 6–8 days in 6-well plates (5 × 10^5^) in the presence of 30 ng/ml granulocyte-macrophage colony-stimulating factor (day 1, 3, 5, R&D Systems). The identity of BMDMs and BMDCs was controlled by staining with fluorescence-labeled antibodies to CD11b (FITC or PE, clone M1/70, BD Biosciences) for BMDMs and to CD11c (FITC or PE, clone HL3, BD Biosciences) for BMDCs and expression analysis by flow cytometry on a BD LSRII flow cytometer. FITC or PE-labeled rat IgG2b, K (A95-1, BD Biosciences) and Armenian hamster (eBio299Arm, eBioscience) isotype controls were used. APCs were cultivated in RPMI 1640 (Sigma) medium supplemented with 10% FCS (Gibco) at 37°C in 5% CO_2_.

### Confocal Microscopy Analysis of Cytosolic Antigen Release

Immortalized mouse macrophages were incubated in the dark for 18 h with 0.2 µg/ml of the photosensitizer TPCS_2a_. Subsequently cells were washed three times with PBS and incubated for additional 4 h with 10 µg/ml 5-Carboxyfluorescein labeled OVA_257–264_ peptide (FAM-OVA_257–264_, Anaspec). Samples were fixed with 2% paraformaldehyde either without light treatment or directly after illumination for 3 min on the LumiSource^®^ light table. Cellular distribution of OVA_257–264_ and photosensitizer TPCS_2a_ fluorescence (27) was analyzed by confocal microscopy on a Zeiss LSM510 confocal microscope with a 63× objective. Excitation at 488 nm and a 505–530 nm band pass filter were used to measure FAM-OVA_257–264_ fluorescence; excitation at 633 nm and a 650 nm long pass filter were used to record emission from TPCS_2a_. Images were processed with Zeiss microscopy software.

### Viability Assay

Bone marrow-derived macrophages, immature BMDCs, and the B6 macrophage cell line were incubated in the dark with 0.2 µg/ml TPCS_2a_ overnight in 96-well plates. Cells were washed three times with PBS and incubated for additional 4 h in TPCS_2a_-free medium. Viability of APCs was assessed 18 h post illumination. For the B6 macrophage cell line, viability was analyzed using the MTT-based CellTiter 96 AQueous One Solution Cell Proliferation Assay (Promega). Absorption at 490 nm was detected on a spectrophotometer. Due to low MTT incorporation, viability of BMDMs and BMDCs was analyzed using the CellTiter-Glo Luminescent Cell Viability Assay (Promega) which quantifies ATP, present in metabolically active cells. Relative light units (RLU) were quantified on a luminometer (PerkinElmer). The assays were performed according to the manufacturer’s protocol. Results were analyzed using GraphPad Prism 5 software (GraphPad Software, Inc.).

### Dendritic Cell (DC) Maturation Assay

Immature BMDCs (5 × 10^5^) were incubated in 6-well plates ± TPCS_2a_ overnight at 37°C, 5% CO_2_ in the dark. TPCS_2a_ was washed (PBS, 3×) from the cells and cells were incubated for additional 4 h before light treatment. Lipopolysaccharide (LPS) from *E. coli* (100 ng/ml, Sigma) was used as a positive control to induce BMDC maturation, untreated cells were used as negative control. 18 h post light treatment, cells were scraped and stained with fluorescence-labeled anti-CD11c (FITC or PE, clone HL3, BD Biosciences), anti-MHC class II (I-Ab, Alexa Fluor 488 or APC, clone AF6-120.1, BioLegend), and anti-CD86 (PE, clone GL1, BioLegend) antibodies. Unlabeled anti-CD16/32 (clone 93, eBioscience) was used to block Fc receptor binding. Flow cytometric analysis of the cells was performed on a BD LSR II flow cytometer (BD Biosciences) and data analysis performed using FlowJo software (v10.0.7, FlowJo, LLC).

### *In Vitro* Antigen Presentation Assays

Immature BMDCs as well as the B6 macrophage cell line were incubated in the dark ±0.2 µg/ml TPCS_2a_ overnight in 24-well plates (1 × 10^5^). TPCS_2a_ was washed away from the cells (PBS, 3×) and the cells were incubated for 4 h in the presence of peptide antigens as indicated. OVA_257–264_ peptide (SIINFEKL), N-terminally extended OVA_248–264_ (9 + SIINFEKL), and C-terminally extended OVA_257–280_ (SIINFEKL + 15) were used for stimulation (all from Anaspec). Cells were illuminated for the indicated periods and incubated overnight in the dark. Cells were stained with anti-CD11c FITC (clone HL3, BMDCs) or anti-CD11b FITC (clone M1/70, B6 macrophage cell line) and anti-H-2Kb-SIINFEKL PE (clone 25-D1.16, eBioscience), specifically recognizing OVA_257–264_ (SIINFEKL) peptide bound to MHC class I (H-2Kb). The anti-H-2Kb-SIINFEKL signal was quantified on CD11c+ cells for CD11b+ cells for the B6 cell line, respectively. Cells were analyzed on a BD LSRII flow cytometer and data analyzed using FlowJo and GraphPad Prism software.

### *In Vitro* T Cell Activation Assays

Bone marrow-derived macrophages, immature BMDCs, or B6 macrophages were incubated in the dark ±0.2 or 0.4 µg/ml TPCS_2a_ overnight in 96-well plates (3 × 10^4^ cells/well). If indicated, 100 ng/ml LPS was added to the culture. Cells were washed (PBS, 3×) and incubated for additional 4 h with 0.01–1 µg/ml OVA_257–264_ peptide. Subsequently, cells were exposed for 0–10 min to blue light on the LumiSource^®^ light table before addition of 1 × 10^5^ OVA_257–264_-specific RF33.70 CD8+ T cell hybridoma cells overnight (kind gift of Kenneth Rock, University of Massachusetts) (28). Supernatants were harvested and IL-2 production from activated RF33.70 cells was analyzed in a bioassay. For this purpose, the IL-2-dependent T cell line HT-2 (ECACC) was grown in medium containing 50% supernatant from RF33.70 T cells. Growth of HT-2 cells was quantified either in a standard ^3^H-thymidine (PerkinElmer) incorporation assay or with the CellTiter-Glo Luminescent Cell Viability Assay (Promega), which quantifies ATP from metabolically active cells. This was due to replacement of the radioactive ^3^H-thymidine assay during the course of the study. Both assays yielded comparable results and similar sensitivity. RF33.70 and HT-2 cells were cultured in RPMI-1640 (Sigma) supplemented with 10% FCS (Gibco), 2 mM l-glutamine (Sigma), 50 µM 2-ME (Sigma), and 20 mM HEPES (Sigma). Data analysis and statistical tests were performed using GraphPad Prism software.

### Animals and *In Vivo* Immunization With PCI Technology

All procedures involving mice experiments were carried out in accordance with institutional guidelines, national legislation, and the Directive of the European Convention for the protection of vertebrate animals used for experimental and other scientific purposes. The protocols were approved by the Norwegian Animal Research Authority. Female C57BL/6 mice were purchased from Harlan and weighed on average 20 g (6–8 weeks old) when experimental procedures were started. Five mice were used in each experimental group.

All peptide antigens that were employed for *in vivo* vaccination were synthesized by United Biosystems (Herndon, VA, USA) with a purity of >98%. The peptides used were amino acids 43–78 from the human papillomavirus (HPV) 16 E7 protein (HPV_43–78_, GQAEPDRAHYNIVTFCCKCDSTLRLCVQSTHVDIR, CD8+ T cell epitope underlined) and amino acids 180–188 from tyrosinase-related protein 2 (TRP-2_180–188_, SVYDFFVWL).

Prior to vaccination, vaccine components were mixed in a volume of 100 µl aqueous solution. The resulting vaccines contained the vaccine peptide antigen ± TPCS_2a_ photosensitizer; no additional adjuvants were used. Mice were shaved on the belly region, and the vaccine solution was injected *intradermally* at two injection sites within the shaved area (50 µl at each site). The mice were kept in the dark. 18 h after immunization, mice were anesthetized by a subcutaneous injection with a mixture giving doses of 10–15 mg/kg xylasin (Rompun^®^, Bayer), 5–10 mg/kg butorphanol (Torbugesic^®^, Zoetis), and 15–20 mg/kg zolazepam and tiletamine (Zoletil^®^, Virbac). Anesthetized mice were positioned on the LumiSource^®^ light table and the vaccination site was exposed to blue light for 6 min. All mice received three identical vaccinations, with 14-day intervals between the treatments. 100 µl blood samples were taken into EDTA tubes 10 days after the last vaccination, mice were sacrificed on day 13 (HPV_43–78_ vaccinated mice) or day 25 (TRP-2_180–188_ vaccinated mice) after the last vaccination to harvest spleen cells.

### Analysis of Antigen-Specific CD8+ T Cell Responses in Vaccinated Mice

Red blood cells were lysed from blood samples of vaccinated mice (RBC lysis buffer, Sigma) and detection of antigen-specific T cells was performed by staining with fluorescent peptide-loaded MHC class I pentamers (ProImmune). Phycoerythrin (PE)-labeled HPV_49–57_ peptide-loaded MHC class I pentamer (H-2Kb/RAHYNIVTF Pro 5 Pentamer) was used to detect antigen-specific CD8+ T cells in HPV_43–78_ peptide vaccinated mice. PE-labeled TRP-2_180–188_ H-2Kb/SVYDFFVWL pentamers were used in TRP-2_180–188_ peptide vaccination experiments. In addition, cells were stained with fluorescent antibodies for CD8 (PerCP-Cyanine5.5, clone 53-6.7, eBioscience) and CD44 (APC, clone IM7, eBioscience). Cells were analyzed on a BD LSRII flow cytometer and statistical tests were performed using FlowJo and GraphPad Prism software. Antigen-specific CD8+ T cells with an activated phenotype were identified from FSC/SSC- and CD8-gated T cells and expression analysis of the activation marker CD44 and binding of the respective peptide-loaded MHC class I pentamer.

Effector cytokine production by CD8+ CTLs was analyzed by intracellular cytokine staining after *ex vivo* restimulation of spleen cells. After lysis of red blood cells, spleen cells were plated in 48-well plates and stimulated with 5 µg/ml antigenic peptide overnight. Brefeldin A (eBioscience) was added for the last 4 h of stimulation. Cell were stained for CD8 (PerCP-Cyanine5.5, clone 53-6.7, eBioscience) and CD44 (PE, clone IM7, eBioscience) before fixation and permeabilization with Intracellular Fixation & Permeabilization Buffer (eBioscience). Intracellular cytokine staining was performed with antibodies to interferon (IFN)-γ (anti-IFN-γ APC, clone XMG1.2, eBioscience) and flow cytometric analysis performed as described above. Frequencies of activated IFN-γ-producing CD8+ effector CTLs cells were identified in the CD8+ CD44+ T cell population.

## Results

### PCI Enables Cytosolic Delivery of Peptide Antigens and Has a Dose-Dependent Cytotoxic Effect on APCs

Photochemical internalization is an innovative technology to route endocytosed molecules of interest to the cytosol of cells by co-endocytosis of cargo material with an amphiphilic photosensitizer molecule that enriches in the membranes of endosomes. Activation of the photosensitizer by illumination induces rupture of endosomal membranes and release of cargo molecules into the cytosol. The treatment primarily affects endosomal membranes where the photosensitizer is enriched (29). But since a fraction of photosensitizer molecules may be located in other membranes, such as the plasma membrane, PCI might have cytotoxic effects on cells. We therefore examined the effect of PCI treatment on the viability of different types of APCs as well as the potential of PCI to route short peptide antigens as cargo to the cytosol of APCs.

**Figure 1:**
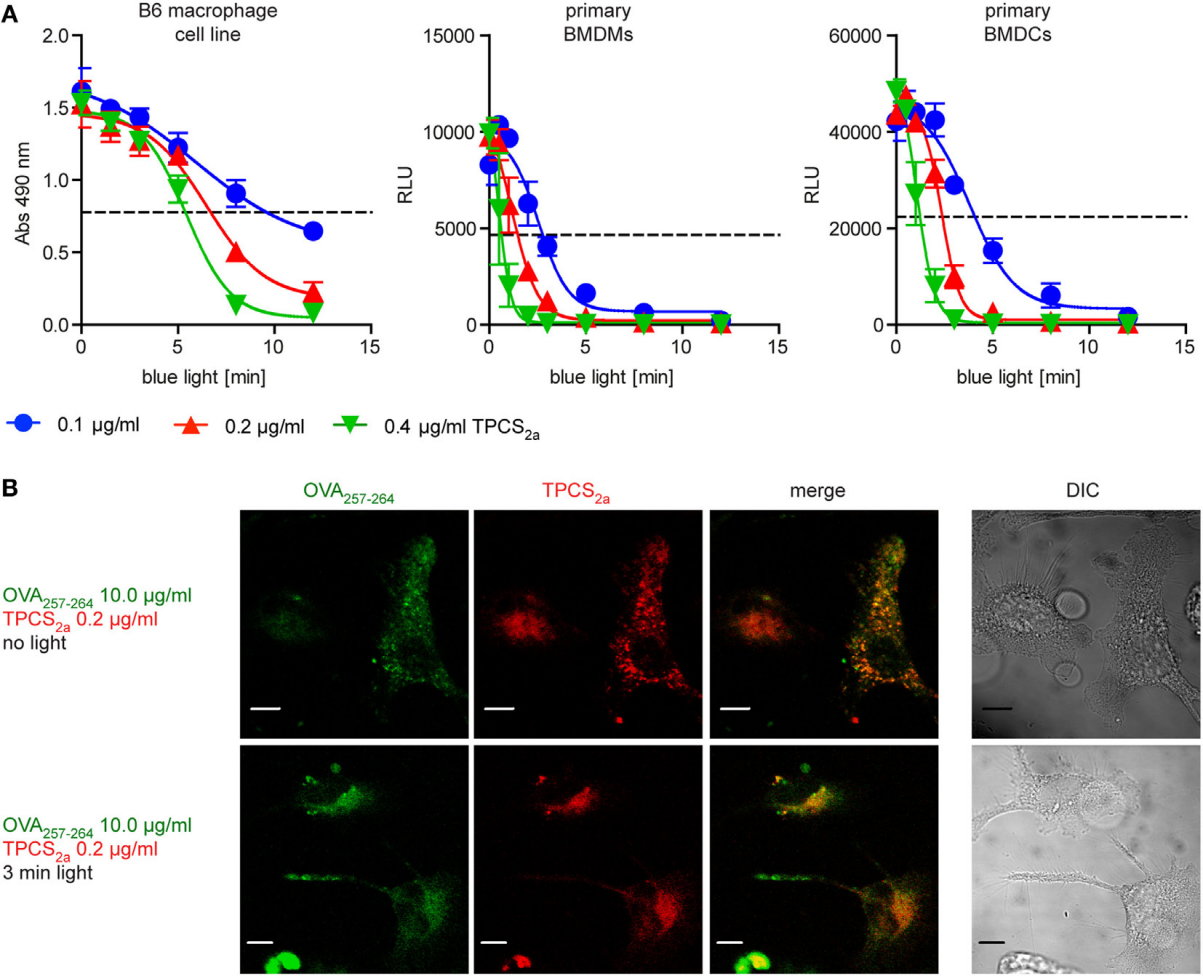
Photochemical internalization (PCI) treatment routes endocytosed peptide antigens to the cytosol of antigen-presenting cells (APCs), though the treatment has a dose-dependent cytotoxic effect. **(A)** Analysis of the effect of PCI treatment on the viability of APCs. Immortalized B6 mouse macrophages (left) as well as primary bone marrow-derived macrophages (BMDMs) (middle) and primary bone marrow-derived dendritic cells (BMDCs) (right) were incubated for 18 h with 0.1 (blue), 0.2 (red), or 0.4 µg/ml (green) of the photosensitizer TPCS_2a_. Cells were illuminated for 0–12 min and viability was assessed 18 h post illumination. The metabolic activity of the B6 cell line was analyzed by an MTT assay (*y*-axis = absorption at 490 nm). Viability of primary BMDMs and primary BMDCs were analyzed by measurement of ATP content in a luminescence assay (*y*-axis = relative light units). The dotted line indicates approximately 50% cell viability. Three or more experiments with comparable results were performed for each cell type, shown is one representative experiment. **(B)** Effect of PCI treatment on the intracellular localization of the photosensitizer TPCS_2a_ and OVA_257–264_ peptide antigen. Immortalized mouse macrophages were incubated in the dark for 18 h with 0.2 µg/ml of the photosensitizer TPCS_2a_. After washing, cells were incubated for additional 4 h with 10 µg/ml 5-Carboxyfluorescein labeled OVA_257–264_ in TPCS_2a_-free medium. The cellular distribution of fluorescence from OVA_257–264_ (red) and TPCS_2a_ (green) was analyzed by confocal microscopy without photo-activation (upper panels) or directly after 3 min blue light illumination (lower panels). Scale bars represent 5 µm.

Possible cytotoxic effects of PCI treatment were tested on an immortalized C57BL/6 mouse macrophages cell line (B6), as well as on primary BMDMs and immature BMDCs from C57BL/6 mice. The impact of PCI treatment on APC viability was tested with three different concentrations of the photosensitizer molecule TPCS_2a_ in combination with photoactivation by increasing illumination periods (Figure 1A). Cells were incubated in the dark with 0.1, 0.2, or 0.4 µg/ml TPCS_2a_ overnight, followed by a 4 h chase period to allow endocytosis of the photosensitizer prior to light exposure of the cells with blue light for 1–12 min, which imposes the photosensitizer to different light doses. Cell viability was analyzed 16 h post light treatment. Treatment with TPCS_2a_ in the dark did not affect the viability of the tested APCs. However, when TPCS_2a_-treated cells were photoactivated all the cell types showed reduced viability, dependent on the illumination time as well as on the photosensitizer concentration. Primary BMDCMs and BMDMCs were found to be more sensitive to PCI treatment compared to the macrophage cell line. With a photosensitizer concentration of 0.2 µg/ml TPCS_2a_ approximately 50% of the B6 macrophage cell line were viable 18 h post light treatment with 5–8 min of blue light (Figure 1A, left), whereas the viability of primary BMDCMs and BMDMCs was reduced to 50% after only 2–3 min illumination (Figure 1A, middle and right). To achieve an optimal effect of PCI on cytosolic release of endocytosed antigen, only a moderate effect of the treatment on the viability of APCs would be tolerable. We therefore used TPCS_2a_ at 0.2 µg/ml for subsequent experiments and focused on illumination periods, where 50% or more of the APCs are assumed to survive.

To study the potential of PCI to direct short peptide to the cytosol of APCs, the antigenic peptide sequence OVA_257–264_ (peptide sequence SIINFEKL), a naturally processed antigenic peptide from ovalbumin that can be presented *via* MHC class I (H-2Kb) in C57BL/6 mice, was used as model antigen. Immortalized C57BL/6 mouse macrophages were incubated in the dark with the photosensitizer TPCS_2a_ and a fluorescence-tagged version of OVA_257–264_ peptide and subsequently photoactivated by illumination with blue light for 3 min (Figure 1B). OVA_257–264_ as well as TPCS_2a_ were endocytosed into macrophages and co-localized in the absence of light to granular structures (Figure 1B, upper panel). We assumed these structures to be endo-lysosomal compartments, as localization of TPCS_2a_ to these compartments has been shown (29). After exposing the cells to light, the fluorescence of both the photosensitizer and fluorescent OVA_257–264_ was found to be distributed more diffusely throughout the cytosol (Figure 1B, lower panel). This indicates that PCI treatment can release endocytosed peptide antigens from endosomes into the cytosol of APCs; in line with previous findings with fluorescence-tagged ovalbumin protein (22, 23).

### PCI Treatment Induces Maturation of Primary Immature DCs

**Figure 2:**
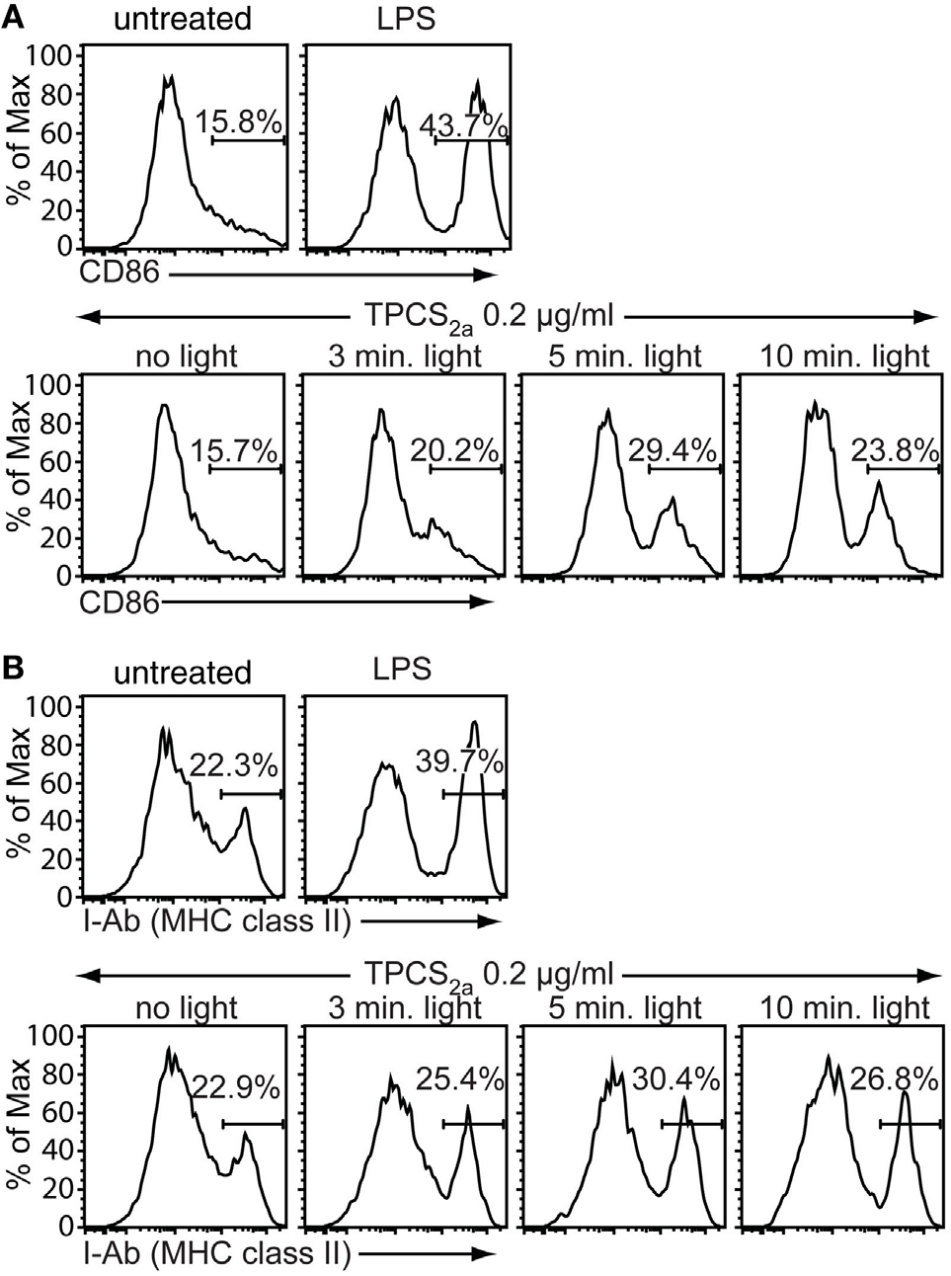
Photochemical internalization treatment promotes maturation of dendritic cells (DCs). Primary immature bone marrow-derived dendritic cells (BMDCs) were incubated for 18 h in the dark with 0.2 µg/ml TPCS_2a_, 100 ng/ml lipopolysaccharide (LPS) or were left untreated. TPCS_2a_-treated cells were washed and incubated in TPCS_2a_-free medium for additional 4 h before illumination for 0, 3, 5, and 10 min with blue light. Expression of the DC maturation markers CD86 **(A)** and MHC class II **(B)** on CD11c-gated BMDCs was analyzed 18 h post illumination by flow cytometry (*y*-axis: % of maximum count). Shown is one representative experiment of three experiments with comparable findings.

Antigen-presenting cells must provide a strong co-stimulatory signal to CD8+ T cells in order to effectively induce CD8+ CTL cell responses. A commonly used way to achieve this is to add APC-activating adjuvants such as toll-like receptor (TLR) ligands or certain cytokines to vaccine formulations. Immature DCs are highly sensitive to danger signals mediated, for example, *via* TLRs, but also to products from cell damage, which might be generated by the photochemical treatment in the PCI procedure. We thus investigated by flow cytometry if the cell damage caused by PCI treatment can enhance expression of the co-stimulatory molecule CD86 and MHC class II on immature BMDCs. Immature BMDCs were treated with 0.2 µg/ml TPCS_2a_ and illuminated for 0–10 min, or stimulated with a TLR4 ligand, LPS, as positive control for induction of DC maturation and activation (Figure 2). TPCS_2a_ treatment in the absence of photoactivation did not affect the expression levels of the activation markers CD86 (Figure 2A) and MHC class II (Figure 2B) on primary BMDCs. However, photoactivation of TPCS_2a_-treated BMDCs resulted in upregulation of both CD86 and MHC class II on the DC surface. The frequency of CD86-expressing BMDCs increased from 16% without light treatment to 30% when BMDCs were illuminated for 5 min. Similarly, the frequency of MHC class II + BMDCs increased from 23% in the absence of light to 30% after 5 min of light treatment. With very long illumination times (10 min), the expression levels of CD86 and MHC class II were reduced, probably due to cytotoxic effects of the PCI treatment. However, the effect of the PCI treatment on DC maturation was still less pronounced than the effect of LPS. These findings indicate that the PCI treatment by itself could provide a direct adjuvant function in therapeutic vaccine regimens.

### PCI-Mediated Routing of Peptide Antigens to the Cytosol of APCs Increases MHC Class I Antigen Presentation

**Figure 3:**
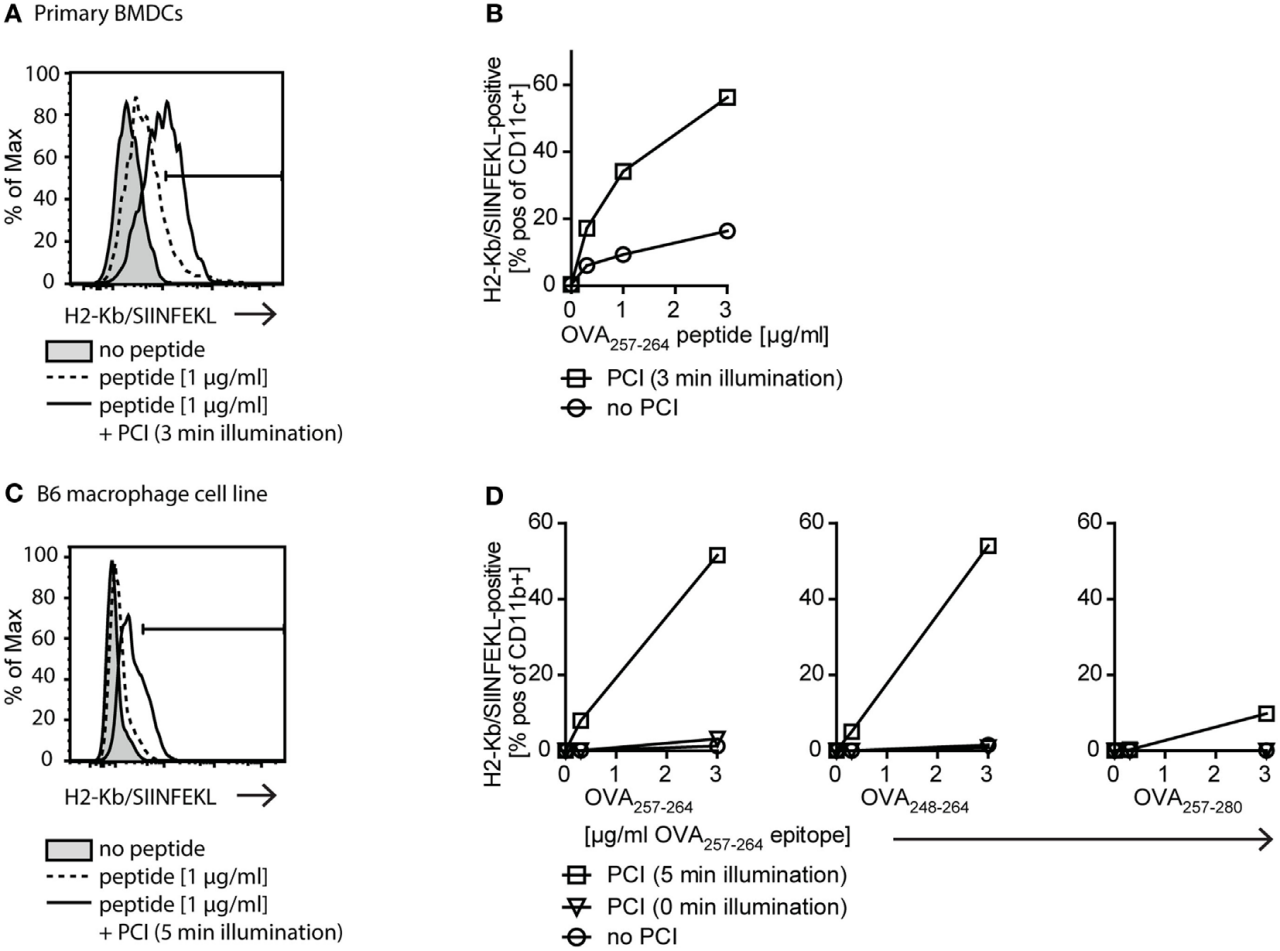
The photochemical internalization (PCI) technology enhances MHC class I antigen presentation. **(A,B)** Effect of PCI on MHC class I antigen presentation on primary bone marrow-derived dendritic cells (BMDCs). Cells were stimulated with 0–3 µg/ml OVA_257–264_ peptide alone or delivered by PCI (0.2 µg/ml TPCS_2a_, 3 min illumination). Presentation of OVA_257–264_ on MHC class I (H2-Kb/SIINFEKL staining) was analyzed by flow cytometry on CD11c+ BMDCs 18 h post illumination. **(A)** Representative example of flow cytometric analysis for stimulation of BMDCs with 1 µg/ml OVA_257–264_ peptide (tinted: no peptide; dotted line: peptide alone; solid line: peptide + PCI). **(B)** Quantification of MHC class I presentation of OVA_257–264_ peptide (H2-Kb/SIINFEKL+ CD11c+ cells) on primary BMDCs stimulated with 0–3 µg/ml OVA_257–264_ peptide with and without PCI. **(C,D)** Effect of PCI on MHC class I antigen presentation on immortalized B6 macrophages. 0–3 µg/ml OVA_257–264_ peptide or N- and C-terminally extended peptides OVA_248–264_ and OVA_257–280_ were delivered to B6 macrophages with or without PCI (0.2 µg/ml TPCS_2a_). PCI was carried out using 5 min illumination, unilluminated TPCS_2a_-treated cells served as control. Antigen presentation on CD11b+ macrophages was analyzed as described above. **(C)** Representative flow cytometry example for stimulation with 1 µg/ml OVA_257–264_ peptide (tinted: no peptide; dotted line: peptide alone; solid line: peptide + PCI). **(D)** Quantification of antigen presentation with and without PCI on B6 macrophages stimulated with 0–3 µg/ml OVA_257–264_ peptide (left) as well as extended OVA_248–264_ (middle) and OVA_257–280_ (right) peptides. Extended peptide concentrations were adjusted to ensure matching concentrations of “SIINFEKL” epitope. Experiments with primary BMDCs and B6 macrophages were repeated three or more times, shown is one representative experiment.

Since we found that PCI treatment of APCs enabled cytosolic delivery of endocytosed antigenic peptides, we next tested if this leads to an increase in MHC class I-restricted cross-presentation of peptide antigen in APCs. To this end, we looked for the presence of the OVA_257–264_ (SIINFEKL) peptide in complex with MHC class I (H-2Kb) on the surface of APCs, which can be detected by flow cytometry using a H-2Kb-SIINFEKL-specific antibody. We investigated MHC class I antigen presentation of OVA_257–264_ with peptide concentrations from 0 to 3 µg/ml with or without PCI treatment on primary BMDCs (Figures 3A,B) and B6 macrophages (Figures 3C,D). The efficiency in MHC class I presentation of OVA_257–264_ antigen was remarkably higher in both, PCI-treated DCs and macrophages, as compared to cells receiving the peptide antigen alone. When OVA_257–264_ peptide was delivered using PCI, an at least 10-fold lower antigen dose was sufficient to elicit antigen presentation compared to incubation with peptide antigen alone. These results show that cytosolic delivery of peptide antigens using PCI technology strongly enhances MHC class I-restricted antigen presentation both in DCs and in macrophages. Off note, mean fluorescence intensities (*x*-axis in the histograms, Figures 3A,C) were found higher in experiments with DCs compared to macrophages, indicative of increased OVA_257–264_/MHC class I presentation on DCs compared to macrophages.

OVA_257–264_ is a short peptide that could be loaded directly onto MHC class I molecules on the surface of APCs. To distinguish between passive loading onto MHC class I and true antigen processing and presentation from the cytoplasmic compartments, we compared antigen presentation of the OVA_257–264_ (SIINFEKL) peptide with presentation of N-terminally extended OVA_248–264_ (9 + SIINFEKL) and C-terminally extended OVA_257–280_ (SIINFEKL + 15) peptide, respectively (Figure 3D). These extended peptides need intracellular processing for correct size and thus, presentation by MHC class I. The N-terminal extension requires non-proteasomal aminopeptidase processing, whereas the C-terminal extension is proteasome dependent (30). We found that the N-extended peptide OVA_248–264_ (9 + SIINFEKL) was presented equally well as the OVA_257–264_ (SIINFEKL) peptide, demonstrating efficient cytosolic delivery and antigen processing using PCI. The presentation of the C-terminally extended peptide OVA_257–280_ (SIINFEKL + 15) was in the highest dose (3 µg/ml) also enhanced by PCI compared to the peptide alone, albeit with a much lower efficiency. Together, these data suggest that PCI-enhanced MHC class I presentation occurs from peptides located in the cytosol of APCs and that non-proteasomal and proteasomal processing mechanisms maybe engaged upon PCI-mediated delivery of extended peptides.

### Delivery of Antigen Using PCI Enhances Antigen-Specific CD8+ T Cell Activation *In Vitro*

**Figure 4:**
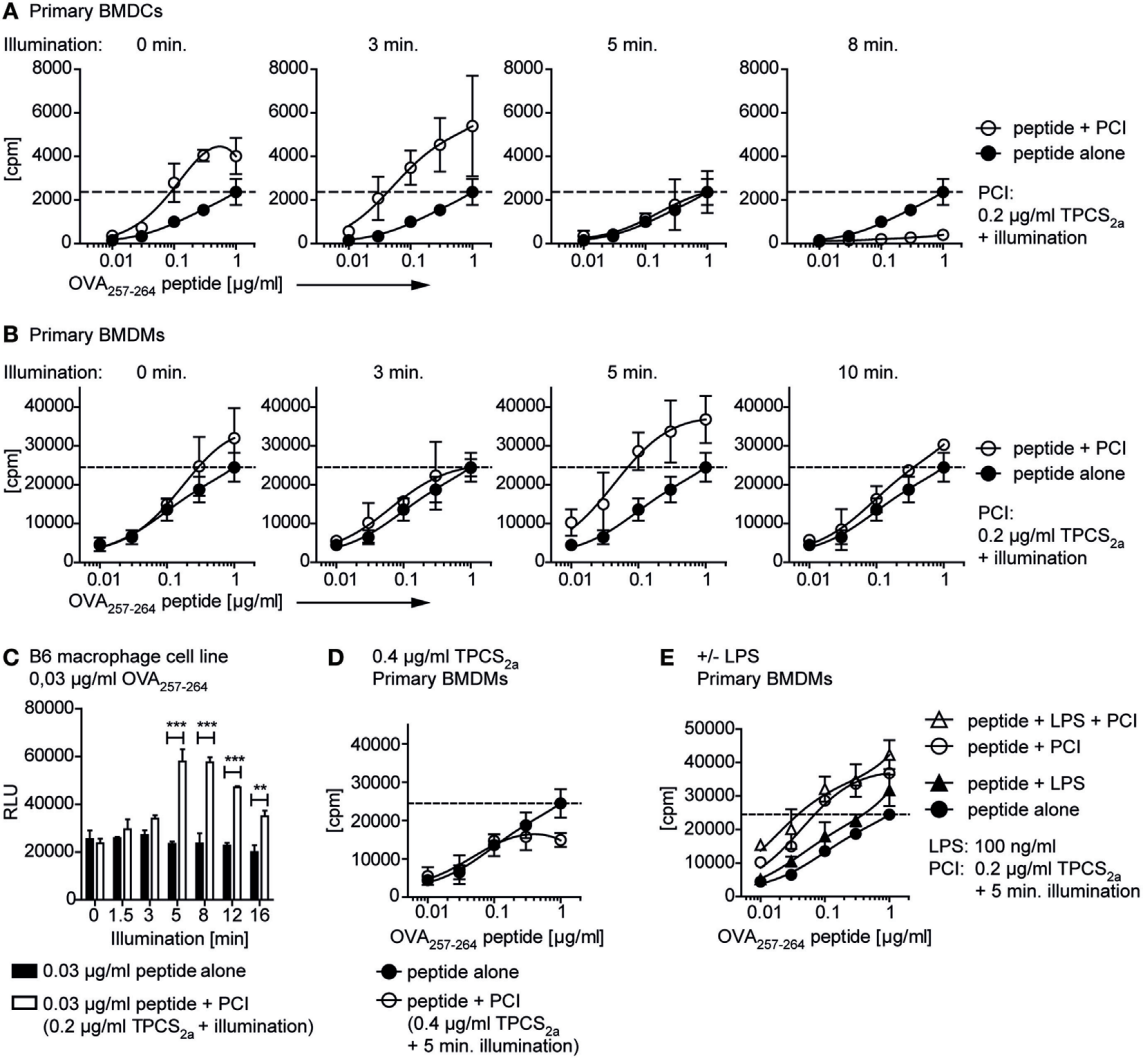
Photochemical internalization (PCI)-mediated peptide antigen delivery to antigen-presenting cells enables CD8+ T cell activation with very low doses of peptide. Primary bone marrow-derived dendritic cells (BMDCs), primary bone marrow-derived macrophages (BMDMs), or the B6 macrophage cell line were incubated ± TPCS_2a_ and OVA_257–264_ peptide as described in Figure 3. Cells were illuminated, before ovalbumin-specific RF33.70 CD8+ T cells were added overnight and IL-2 from activated CD8+ T cells was analyzed in a bioassay from cell culture supernatants. The PCI-effect on RF33.70 CD8+ T cell activation with 0.01–1 µg/ml OVA_257–264_ OVA_257–264_ peptide was analyzed in primary BMDCs **(A)** and primary BMDMs **(B)**. The graphs show results for CD8+ T cell activation with peptide alone (closed symbols) or peptide delivered by PCI (0.2 µg/ml TPCS_2a_, open symbols) and increasing illumination times from 0 to 10 min. The dotted line indicates the maximal CD8+ T cell activation achieved with 1 µg/ml OVA_257–264_ peptide without PCI treatment. **(C)** Effect of PCI treatment on OVA_257–264_-specific CD8+ T cell activation with a single, low concentration of 0.03 µg/ml OVA_257–264_ peptide and increasing illumination times. OVA_257–264_ peptide was delivered to the B6 macrophage cell line without (black bars) and with PCI (0.2 µg/ml TPCS_2a_, 18 h, white bars) and illuminated for 0–16 min. Experiments were performed in triplicates; *P* values for each illumination time were calculated using an unpaired two-tailed *t*-test (*P < 0.05, **P < 0.01, and ***P < 0.005). **(D)** Effect of PCI treatment with a high dose of TPCS_2a_ (0.4 µg/ml) on OVA_257–264_-specific CD8+ T cell activation. BMDMs were incubated with 0.01–1 µg/ml OVA_257–264_ peptide alone (open symbols) or peptide delivered by PCI using 0.4 µg/ml TPCS_2a_ and 5 min illumination. **(E)** Effect of additional toll-like receptor stimulation. BMDMs were treated ± lipopolysaccharide (LPS) (100 ng/ml), with or without PCI treatment (0.2 µg/ml TPCS_2a_, 5 min illumination). The bioassay measured proliferation [^3^H-thymidine incorporation in **(A,B,D,E)**; cpm, counts per minute] or metabolic activity [CellTiter-Glo Luminescent assay in **(C)**; RLU, relative light units] of the IL-2-dependent cell line HT-2 grown in RF33.70 cell culture supernatant. The method was changed due to replacement of the radioactive ^3^H-thymidine assay during the study. Graphs represent mean values ± SD, experiments were performed in triplicates and repeated three times.

We next investigated whether PCI-mediated cytosolic peptide delivery can enhance antigen-specific CD8+ T cell activation *in vitro* using OVA_257–264_-specific CD8+ T cells. The dose–response effects of the PCI peptide delivery strategy was studied by varying the doses of peptide, photosensitizer, and illumination time in macrophages and DCs (Figure 4). APCs were preloaded for this purpose with increasing concentrations of OVA_257–264_ peptide (0–1 µg/ml) with or without TPCS_2a_. Subsequently cells were illuminated (0 - 16 min) and co-cultured with RF33.70 OVA_257–264_-specific CD8+ T cell hybridoma cells. Stimulation of primary BMDCs (Figure 4A) and primary BMDMs (Figure 4B) with OVA_257–264_ peptide alone, resulted in a peptide concentration-dependent CD8+ T cell activation (closed symbols in the panels). At a fixed dose of 0.2 µg/ml TPCS_2a_ (open symbols in the panels), PCI induced an illumination-dependent change in CD8+ T cell activation. At the optimal light dose of 3 min illumination for primary BMDCs, approximately 20-fold more efficient antigen presentation was observed in PCI-treated cells than what was found with peptide antigen alone (Figure 4A). For primary BMDMs, a slightly higher light dose (5 min) seemed optimal to maximize antigen presentation; a 20-fold enhancement was obtained by PCI treatment (Figure 4B), similar to what was seen with the BMDCs. At longer illumination times, the PCI-enhancing effect on CD8+ T cell activation diminished in both macrophages and DCs. Thus, at the highest light dose tested (10 min), the CD8+ T cell activation using DCs was totally abrogated, possibly due to increased death of the APCs (see also Figure 1). It is of note that in the BMDCs, TPCS_2a_ alone (i.e., with no illumination) also seemed to increase CD8+ T cell activation compared to the control. We further characterized the PCI effect at very low peptide antigen doses, below the level where CD8+ T cell activation could be detected with peptide alone. Thus, we incubated the macrophage B6 cell line with OVA_257–264_ peptide at 0.03 µg/ml with or without 0.2 µg/ml TPCS_2a_ and activated the photosensitizer with light doses from 0 to 16 min before addition of OVA_257–264_-specific CD8+ T cells (Figure 4C). Under these conditions, illumination periods of 5 min and more resulted in a strong and highly significant increase in CD8+ T cell activation, which again decreased with illumination times of more than 12 min.

Our findings show that with optimal combinations of antigen, photosensitizer, and light, *in vitro* CD8+ T cell activation can be increased about 20 times as compared to what is achieved with peptide antigen alone. Using 0.2 µg/ml TPCS_2a_, we usually found that 2–3 min light treatment was most effective to activate CD8+ T cells with primary BMDCs, whereas 5–8 min illumination seemed optimal for BMDMs. No beneficial effect on CD8+ T cell activation from BMDMs was observed by doubling the amount of photosensitizer treatment (0.4 µg/ml TPCS_2a_, 5 min illumination, Figure 4D). These results may indicate that too much of either component in the system, illumination time or photosensitizer dose, may abrogate the positive effects on CD8+ T cell activation, possibly due to enhanced cytotoxic effects.

To study the effect of LPS as additional adjuvant for activation of antigen-specific CD8+ T cells, we repeated the experiments with primary BMDMs ± PCI (±0.2 µg/ml TPCS_2a_ with 5 min illumination) in the presence or absence of LPS (Figure 4E). We found that stimulation with OVA_257–264_ antigen and LPS alone increased *in vitro* CD8+ T cell activation compared to antigen alone, although rather moderately as compared to the 20-fold increase achieved by using PCI without LPS. Thus, this experiment clearly showed a superior efficiency of the PCI approach for antigen-specific CD8+ T cell activation as compared to the more common approach of co-administrating peptide and a TLR ligand for immunization properties. We did not see a clear benefit on CD8+ T cell activation from combining PCI treatment with adding LPS as adjuvant to further stimulate antigen presentation (Figure 4E).

### PCI Facilitates Activation of Naïve Antigen-Specific CD8+ T Cells With Adjuvant-Free Peptide Antigen *In Vivo* in C57BL/6 Wild-Type Mice

It has recently been shown that *intradermal* vaccination delivering ovalbumin protein with PCI technology facilitates activation of ovalbumin-specific CD8+ T cells that were adoptively transferred from T cell receptor transgenic mice (22, 23). For further development of PCI as a vaccination enhancement technology it is crucial to show that PCI is able to prime naïve endogenous CD8+ CTLs cells *in vivo* using clinically relevant peptide antigens. The antigenic peptides employed in these experiments originate from HPV 16 E7 protein (HPV_43–78_ peptide) and TRP-2_180–188_ peptide of which HPV human peptide homologs have been used in clinical therapeutic cancer vaccination studies (31, 32).

**Figure 5:**
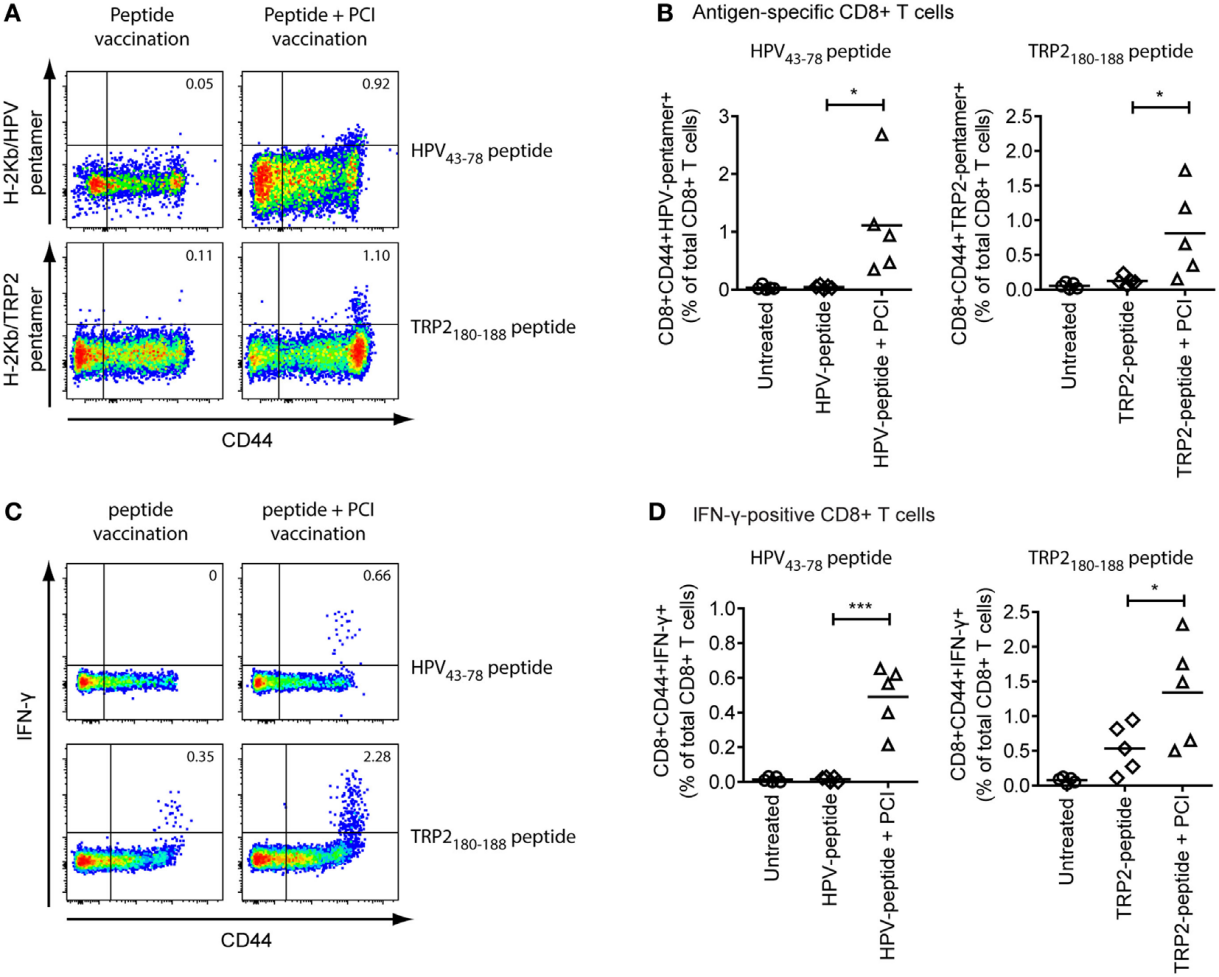
*Intradermal* vaccination of C57BL/6 wild-type mice with peptide antigens and photochemical internalization (PCI) effectively induces antigen-specific CD8+ effector T cell responses. Groups of five C57BL/6 wild-type mice were *intradermally* immunized with TRP-2_180–188_ or HPV_43–78_ peptide ± PCI treatment. Mice received three identical vaccination treatments with 14-day intervals. **(A)** 10 days after the last vaccination, frequencies of activated, antigen-specific CD8+ CTLs were analyses from blood samples with HPV_49–57_ or TRP-2_180–188_ peptide-loaded MHC class I pentamers (CD8+ CD44+ pentamer+ cells). Plots show representative examples of flow cytometric analysis for HPV_43–78_ (upper panels) or TRP-2_180–188_ (lower panels) vaccinated mice without (left) or with PCI treatment (right). **(B)** Quantification of activated HPV_43–48_-specific (CD8+ CD44+ H-2Kb/HPV_49–57_-pentamer+ cells, left) and TRP-2_180–188_-specific (CD8+ CD44+ H-2Kb/TRP-2_180–188_-pentamer+ cells, right) activated CD8+ T cells from all five mice in each group of the experiment. **(C)** Frequencies of interferon (IFN)-γ effector cytokine producing CD8+ CTLs were analyzed from spleen cells after overnight stimulation with TRP-2_180–188_ or HPV_43–78_ peptide, respectively (CD8+ CD44+ IFN-γ+ cells). Plots show representative examples of flow cytometric analysis for HPV_43–78_ (upper panels) or TRP-2_180–188_ (lower panels) vaccinated mice without (left) or with PCI treatment (right). **(D)** Quantification of IFN-γ effector cytokine production from CD8+ T cells (CD8+ CD44+ IFN-γ+ T cells) from HPV_43–48_-vaccinated (left) and TRP-2_180–188_-vaccinated mice for all five mice in each group of the experiments. Horizontal lines in scatter graphs indicate median values; *P* values were calculated using an unpaired two-tailed *t*-test to compare pairs of datasets (*P < 0.05). Vaccination experiments were performed twice and with similar results.

Mice received three *intradermal* vaccinations with the HPV_43–78_ or TRP-2_180–188_ peptides alone or in combination with TPCS_2a_. For PCI treatment, mice were illuminated for 6 min with blue light 18 h post immunization. Antigen-specific CD8+ CTL activation and IFN-γ effector cytokine production were analyzed by flow cytometry in blood cells and splenocytes after the last vaccination (Figure 5). Antigen-specificity of CD8+ CD44+ activated T cells was assessed by additional staining with peptide-loaded fluorescent MHC class I pentamers (Figures 5A,B). IFN-γ production from activated CD8+ CD44+ T cells was analyzed by intracellular cytokine staining of splenocytes after overnight stimulation with antigenic peptide (Figures 5C,D). We found that neither of the peptide antigens alone did induce activation of a clearly detectable antigen-specific CD8+ T cell response (as detected by CD8+ CD44+ Pentamer+ T cells). However, PCI treatment strongly increased the number of HPV-specific CD8+ T cells, with >10-fold increases in the median percentage of HPV-specific CD8+ T cells compared to vaccination with the peptide alone. Likewise, a substantial (>6-fold) increase in the number of TRP-2-specific activated CD8+ T cells was seen when mice were immunized with TRP-2 peptide plus PCI treatment. For IFN-γ production in spleen cells, a 26-fold increase was observed with the HPV_43–78_ peptide in combination with PCI, as compared to vaccination with the peptide alone (Figures 5C,D). Vaccination with the TRP-2_180–188_ peptide alone induced some IFN-γ effector response from activated CD8+ T cells, although a 2.2-fold increase in IFN-γ production was seen when the TRP-2 peptide was administered using PCI. This cancer antigen-specific IFN-γ effector cytokine production indicates activation of functional CD8+ effector T cells by PCI vaccination.

Taken together our results show that the PCI-enhanced MHC-I antigen presentation and DC activation observed *in vitro* efficiently translates into a substantial improvement of cancer peptide antigen vaccination in normal mice *in vivo*.

## Discussion

The use of patient-specific peptide antigens is a very attractive approach for therapeutic tumor vaccination. Peptides in clinical grade quality can be quickly and economically synthesized based on tumor sequencing data, which makes personalized tumor vaccination a viable possibility in the near future. However, priming of tumor-specific CD8+ CTLs, thought to be central for effective anti-tumor immunity, is a challenging task with peptide antigens. Here, we show evidence that the use of PCI technology to deliver peptide antigens to the cytosol of APCs can effectively enable priming of antigen-specific CD8+ CTL responses. PCI technology may thus provide a novel approach toward realization of the large clinical potential of therapeutic cancer vaccination.

In the last few years, immunotherapy with checkpoint inhibitors has in many ways revolutionized the treatment of cancer, with strong and durable responses observed in several important cancer indications. There is, however, a large fraction of patients who do not respond properly to such therapy. This is probably because these patients have not raised or maintained an immune response to the tumor cells that can be exploited as the basis for the immunotherapy. Recent studies have indicated that the response to checkpoint inhibitor immunotherapy is strongly linked to the mutational load of tumors (33) and that tumor neo-epitopes probably are of prime importance for the body to raise an immune response to the tumor cells [reviewed in Ref. (34, 35)]. The effect of cancer immunotherapy could thus be substantially improved by therapeutic vaccination in order to prime immune responses to neo-epitopes found in tumors from individual patients. However, despite some recent encouraging results in cancer vaccine development (9, 36), peptide-based vaccination has so far mostly not been successful in humans, with a lack of efficacy observed in all major clinical studies with this approach (10).

Photochemical internalization technology [originally described in Ref. (11), reviewed in Ref. (13, 37)] represents an innovative approach to promote cytosolic antigen delivery, where a photosensitizing compound permeabilizes membranes of endocytic vesicles upon light activation and releases co-endocytosed antigen into the cytosol. This principle has been demonstrated to successfully induce CD8+ CTL responses to a protein antigen in a mouse model system with adoptively transferred T cell receptor transgenic CD8+ T cells (21). In contrast to, for example, the use of pH-sensitive liposomes (38), the PCI technology enables the release of antigen from endosomes before initiation of acidification and extensive lysosomal degradation. This may be important for effective subsequent MHC class I antigen presentation (39).

Antigens presented on MHC class I usually pass through the cytosol of APCs where they are processed by the proteasome and different aminopeptidases. They are then transported into the lumen of the reticulum where they are trimmed and loaded onto MHC class I molecules for presentation to CD8+ T cells. Infection of APCs by live viral or bacterial vaccines can give rise to cytosolic antigens and MHC class I presentation, but this is much more difficult to achieve with vaccines based on subunits or recombinant proteins, let alone with peptide-based vaccines. Upon endocytosis, peptides have no means to escape from the endocytic vesicles and subsequently face lysosomal degradation or enter the MHC class II antigen presentation pathway with the result of CD4+ T helper cell activation. Translocation of a fraction of endocytosed antigen into the cytosol can naturally occur in DCs in a process termed cross-presentation by which endocytosed antigens may access the MHC class I antigen presentation machinery [reviewed in Ref. (40)]. However, in most cases naturally occurring cross-presentation in DCs seems not to be sufficient to effectively induce CD8+ CTL responses toward endocytosed vaccine antigens. Exploitation of subunit, protein, or peptide vaccines for priming of CD8+ CTL responses therefore requires development of delivery systems that allow delivery of antigens into the cytosol of APCs, from where the antigen can access the MHC class I antigen presentation machinery (8). Translocation of antigen from endosomes to the cytosol of APCs is still challenging, despite many efforts through a variety of different approaches, including new adjuvant strategies (41) as well as the use of cell penetrating peptides (42) and liposomal (38, 43) or biodegradable microsphere vaccine formulations (44, 45).

In the present work, we show that PCI technology can induce the release of endocytosed short peptide antigens into the cytosol of APCs, in accordance with what has been previously described for protein antigen (21, 22). Using an antibody specifically recognizing MHC class I/OVA_257–264_ complexes we were able to visualize that PCI strongly enhances MHC class I cross-presentation of peptide antigens. This was found both when vaccinating with the short OVA_257–264_ peptide itself, as well as with N- or C-terminally extended versions of this peptide. Thus, we found up to 20-fold increased MHC class I/OVA_257–264_ presentation on APCs compared to what was achieved with the peptide antigens alone. Our *in vitro* results with the OVA_257–264_ peptide and *in vivo* results with the TRP-2_180–188_ peptide showed that PCI-mediated delivery of short peptides, that do not require additional proteolytic processing, can effectively enhance MHC class I antigen presentation and induce CD8+ T cell activation. This is interesting, since these short peptides might possibly be directly loaded onto MHC class I molecules on the APC surface by exchange of other MHC class I-bound peptides. Our results suggest that direct peptide loading onto MHC class I molecules on the surface of APC seems inefficient, compared to cytosolic delivery of peptide using PCI. This is in accordance with proposed mechanisms for peptide loading onto MHC class I, where nascent MHC class I molecules in the endoplasmic reticulum are assisted by a protein complex to facilitate peptide loading, eventually also enabling loading of peptides with suboptimal MHC class I fitting properties [reviewed in Ref. (39)].

In addition, the experiments with N- or C-terminally extended peptides of the OVA_257–264_ motif showed that PCI-mediated cytosolic peptide delivery was feeding into both the proteasome-dependent and -independent antigen presentation pathways. These steps may be rate limiting for antigen presentation, since we found less efficient PCI-mediated presentation for C-terminally extended OVA_257–280_, which requires proteasomal processing prior to presentation. In previous work with a protein antigen, it was also found that PCI-enhanced antigen presentation was proteasome dependent (23), though the exact molecular mechanism and localization of the peptide processing in PCI, in particular for peptides, warrants further studies. PCI-enhanced proteaseome-independent presentation was found with the short OVA_257–264_ peptide and with an N-terminally extended peptide requiring processing by aminopeptidases (23).

Since peptide antigens themselves are poorly immunogenic, they generally need the help of additional immunostimulatory signals in order to prime an immune response. Thus, several immunostimulatory compounds that activate TLR signaling pathways are currently tested as possible peptide vaccine adjuvants in animal studies as well as in pre-clinical and clinical studies (46–48). It has earlier been indicated that PCI can increase cytokine production from DCs *in vitro*, in particular interleukin-6 (21). In the present work, we found that PCI treatment of immature DCs induces upregulation of maturation markers and co-stimulatory molecules, such as MHC class II and CD86. These findings indicate that, in addition to the strong enhancement of MHC class I antigen presentation, PCI treatment may have the benefit of an adjuvant effect on immature DCs. As a unique mode of action, this adjuvant effect may be mediated by a low-grade cell damage induced by PCI treatment in APCs, which may trigger endogenous danger signaling and activation of DCs. Similar effects have also been demonstrated in previous studies on photodamage-induced immunogenic cell death [reviewed in Ref. (49)]. We found primary cells more sensitive to cell death than the tested murine macrophage cell line, and especially BMDCs seemed to be sensitive to photochemically induced cell death. The effect of PCI on DC maturation was less pronounced than the effect of the TLR4 ligand LPS. Nonetheless, PCI treatment activated antigen-specific CD8+ T cells more effectively than LPS in our *in vitro* studies, probably since LPS lacks the capacity of cytosolic antigen delivery. However, we did not observe a clear additional benefit of adding LPS to the PCI treatment, though there is a clear possibility that the combination of PCI with other types of TLR ligands or immunological adjuvants could give synergistic effects, and experiments exploring such combinations are ongoing.

For the optimal use of PCI in vaccination, it is crucial to develop balanced *in vivo* immunization protocols that can exploit both the antigen presentation effect and the adjuvant effect of PCI without causing excessive cell damage. When delivering the OVA_257–264_ peptide using PCI, we found in our *in vitro* experiments that 10- to 100-fold lower antigen doses were sufficient to achieve the same CD8+ T cell stimulating effect as with the peptide alone. This was seen with both, primary DCs and macrophages as APCs, but seemingly, DCs required PCI treatment with lower illumination times than macrophages for an optimal effect. DCs enabled increased CD8+ T cell activation even in the absence of photoactivation. The reason for this is unknown, but could possibly be ascribed to stray light reaching the cells during the experiment, in accordance with the finding that DCs seemed to be more sensitive to PCI treatment than macrophages. With increased photosensitizer dose or illumination time, the positive effect of PCI-mediated peptide delivery on CD8+ T cell stimulation was abrogated or even inhibited. A possible reason for this might be that increased photosensitizer doses or illumination times are too cytotoxic to enable good CD8+ T cell activation as the photochemical effect will be a function of the product of the photosensitizer concentration and the light dose. A dose-dependent cytotoxic effect of PCI on APCs has also been previously described (21). Although killing of the target APCs is clearly not desired, PCI might trigger inflammatory and danger signaling pathways such as TLR signaling in cells that are only modestly affected. This may result in APC maturation with upregulation of co-stimulatory molecules and thus contribute to the adjuvant effect of PCI. In addition, surviving APCs might sense endogenous inflammatory signals that are released from neighboring cells that undergo cell death due to the treatment. The optimal effect of PCI-enhanced CD8+ T cell activation was found to be different between DCs and macrophages, as illumination with 3 min or less was optimal for DCs, but for macrophages five or more minutes was found optimal to achieve a beneficial effect on CD8+ T cell activation.

For *in vivo* vaccination, the antigen and the photosensitizer will usually be administered by local injection followed by illumination of the injection site. With such a procedure, cells in the illuminated area will be subjected to gradients both of photosensitizer concentration (depending on the distance from the injection site) and of light dose (depending on how deep in the tissue the APCs are located). A fraction of the APCs in the vaccination area will thus receive an optimal combination of photosensitizer, light and antigen and activation; and these APCs may be sufficient to prime a CD8+ CTL immune response, although other APCs may receive suboptimal or even toxic doses. Since developing the PCI technology for therapeutic cancer peptide vaccination is an important goal, we performed *in vivo* studies with two cancer-relevant peptide antigens to show that the technology is able to effectively prime CD8+ CTL responses from the endogenous T cell repertoire. Our results clearly showed that PCI technology could effectively induce and significantly enhance antigen-specific CD8+ CTL responses to both peptide antigens. In earlier studies, a similar effect has been demonstrated with the ovalbumin protein antigen in mice adoptively transferred with OVA_257–264_-specific transgenic T cells (21–23, 50, 51). However, the results in the present work are the first demonstration of priming of CD8+ T cells from the endogenous T cell pool with clinically relevant peptide sequences, moving the technology substantially closer to a clinical use for peptide vaccination in humans. In addition, our results indicate that cancer-specific CD8+ CTL responses induced by PCI vaccination are functional, as we found IFN-γ effector cytokine production upon *in vitro* restimulation with cancer antigen. Further *in vivo* studies will be necessary to test the effectiveness of PCI vaccination with further cancer-related antigens and tumor neoantigens, as well as to investigate functionality of CD8+ CTLs in more detail, for example, by analyzing the effect on lysis of tumor cells.

Taken together the results of the present work show that the PCI technology using TPCS_2a_ has a large potential for enhancing the effect of peptide-based vaccinations both by increasing MHC class I antigen presentation and by a possible adjuvant effect of the photochemical treatment. For several reasons TPCS_2a_ is a very convenient molecule for the use in clinical vaccine applications: (i) in the absence of photoactivation TPCS_2a_ generally has low systemic and local toxicity; (ii) TPCS_2a_ can be autoclaved and is stable in solution at room temperature for several years; and (iii) the substance (administered systemically) has already been tested with promising results in several clinical trials for local enhancement of the effect of cytotoxic drugs in cancer therapy (20).

Although the focus of the present work has been on therapeutic cancer vaccination, the effect of PCI can potentially be of value also for therapeutic vaccination against infections by viruses and intracellular bacteria, and also for prophylactic vaccination for diseases where an improved CTL immune response would be of value. Work is ongoing for optimizing the PCI vaccination regimen and for identifying possible synergistic combinations of PCI with adjuvants, vaccine delivery systems, and immunotherapeutic agents. A clinical phase I study in healthy volunteers has recently been started to study the safety, tolerability, and effect of *intradermal* PCI-based vaccination.

## Ethics Statement

This study was carried out in accordance with institutional guidelines, national legislation, and the Directive of the European Convention for the protection of vertebrate animals used for experimental and other scientific purposes. The protocols were approved by the Norwegian Animal Research Authority.

## Author Contributions

MHa designed and performed experiments, analyzed and interpreted data, and wrote the manuscript. GB, OG, and TF contributed to *in vitro* experiments, ØH performed confocal microscopy experiments. MHå, AN, and VE performed and analyzed *in vivo* vaccination experiments. ØH, TF, PS, and AH interpreted data and contributed to writing and editing of the manuscript. ØH, PS, and AH conceived the study and overviewed the design and interpretation of results.

## Conflict of Interest Statement

AH and VE are employees of PCI Biotech AS. AH owns shares in the company and is inventor of patents owned by PCI Biotech AS describing the use of PCI in immunization and vaccination. The other authors declare no conflict of interest.

## Abbreviations

APC, antigen-presenting cell; B6, immortalized C57BL/6 mouse macrophage cell line; BMDC, bone marrow-derived dendritic cell; BMDM, bone marrow-derived macrophage; CTL, CD8+ cytotoxic T lymphocyte; DC, dendritic cell; HPV, human papillomavirus; IFN-γ, interferon-γ; OVA, ovalbumin; PCI, photochemical internalization; TPCS_2a_, meso-tetraphenyl chlorin disulphonate, fimaporfin; TRP-2, tyrosinase-related protein 2.
